# Molecular characterisation of human penile carcinoma and generation of paired epithelial primary cell lines

**DOI:** 10.1002/1878-0261.70156

**Published:** 2025-11-25

**Authors:** Simon Broad, Mahmood Hachim, Tom Bertin, Karolina Penderecka, Rifat Hamoudi, Saif Khan, Rui Henrique, Natalie Bergmoser, Manit Arya, Kalle Sipila, Matteo Vietri Rudan, Asif Muneer, Aamir Ahmed

**Affiliations:** ^1^ Centre for Stem Cell and Regenerative Medicine King's College London UK; ^2^ College of Medicine Mohammed Bin Rashid University of Medicine and Health Sciences Dubai United Arab Emirates; ^3^ Research Institute for Medical and Health Sciences, College of Medicine University of Sharjah United Arab Emirates; ^4^ Research Department of Surgical Biotechnology, Division of Surgery and Interventional Science University College London UK; ^5^ ASPIRE Precision Medicine Research Institute Abu Dhabi University of Sharjah United Arab Emirates; ^6^ Research Department of Pathology University College London UK; ^7^ Department of Pathology Portuguese Oncology Institute Porto Portugal; ^8^ Department of Pathology and Molecular Immunology, Abel Salazar Institute of Biomedical Sciences University of Porto Portugal; ^9^ Princess Alexandra Hospital Harlow UK; ^10^ Department of Urology and National Institute of Health Research, Biomedical Research Centre University College London Hospital and Division of Surgery and Interventional Sciences London UK; ^11^ Department of Cell and Developmental Biology, The Centre for Cell and Molecular Dynamics, Rockefeller Building University College London UK; ^12^ Present address: Department of Metabolism Digestion and Reproduction, Commonwealth Building, Hammersmith Campus, Imperial College London UK; ^13^ Present address: Division of Cell Matrix Biology and Regenerative Medicine University of Manchester Manchester UK

**Keywords:** cell lines, rare cancer, urology, Wnt signalling

## Abstract

Penile carcinoma is a rare malignancy in developed countries but is more common in South America and East Africa. The small number of cases means there are limited resources to investigate disease pathogenesis. This report describes a method of generating primary cell lines from freshly isolated human penile tissue using a clonal expansion approach on mitotically inactivated fibroblasts. Matched normal and penile cancer cell lines from two patients were generated and characterised. Molecular karyotyping and targeted sequencing were performed to compare their genomic landscape. Gains in 8q13.3, 10q23.2, 10q25.1, 10q26.13,12p13.33, 20q13.33, Xq21.1 or losses in Yq11.23 were consistent in both tumour cell lines. Gains in 8q13.3 and Xq21.1 cytobands positively correlated with changes in the expression of nearby genes. The top 20 differentially expressed genes are involved in immune responses like interferon alpha/beta signalling. Additionally, there was an increase in integrin β1, transglutaminase 1, keratins 5, 10, 14 and 16, and a decrease in involucrin protein expression. The cell lines described in this study can provide an invaluable platform for new insights and testing of therapies for penile carcinoma.

## Graphical abstract

Generation of two normal and tumour (cancerous) paired human cell lines using an established tissue culture technique and their characterisation is described. Cell lines were characterised at cellular, protein, chromosome and gene expression levels and for HPV status. These cell line may serve as useful *in vitro* tool for the investigations of penile cancer, a rare and neglected disease.

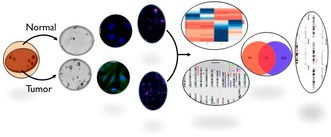

**Abbreviations**
BP: biological process
CC: cellular component
CL2: containment level 2
CN: copy number
CNV: copy number variation
CSC: cancer stem cells
CSPG: chondroitin sulfate proteoglycans
DAPI: 4',6‐diamidino‐2‐phenylindole
DEG: differentially expressed genes
DMEM: Dulbecco's modified Eagle's medium
DMSO: dimethyl sulfoxide
DSPG: dermatan sulfate proteoglycans
E‐Cad: E‐cadherin
ECM: extracellular matrix
EGFR: epidermal growth factor receptor
EYA1: Transcriptional Coactivator And Phosphatase 1
GO: Gene Ontology
HPV: Human papilloma virus
HPV‐HR/LR: HPV high risk/low risk
HSPG: heparan sulfate proteoglycan
ITGB1: integrin β1
IVL: involucrin
K5, ‐10, ‐14, ‐16: keratin 5, ‐10, ‐14, ‐16
KEGG: Kyoto Encyclopedia of Genes and Genomes
MF: molecular function
N: normal
p16INK4A: INK4 family member p16 cell cycle regulator
PBS: phosphate‐buffered saline
PeIN: penile intraepithelial neoplasia
PSCC: penile squamous cell carcinoma
QC: quality control
RNA‐seq: RNA sequencing
RT: room temperature
SCC: squamous cell carcinoma
SD: standard deviation
SNP: single nucleotide polymorphism
STR: short tandem repeat
T: tumour
TGM1: transglutaminase 1
UCL: University College London

## 1 Introduction

Penile cancer is a rare genital malignancy that predominantly affects the foreskin and glans of the penis [1]. Histologically, squamous cell carcinoma (SCC) is the commonest subtype and accounts for more than 95% of the reported cases [2] affecting men, largely, between 50 and 70 years. Although rare in developed countries (accounting for about 1% of all malignancies in men), it is more prevalent in South America, Asia and Africa [3]. In some Asian, African, and South American countries, penile cancer can represent up to 10% of malignancies; for example, in Paraguay and Uganda, the incidence rates are 4.2 and 4.4 per 100 000, respectively [4]. Contemporary therapeutic options for primary cancer are limited to surgical resection of the tumour with either part of the penis (penile‐preserving procedures) or partial or total penectomy in more advanced cases. Radiotherapy or chemotherapy is not recommended as a primary therapeutic option [5], and there is a shortage of research material available to investigate and develop novel targeted therapies.

Penile squamous cell carcinoma is considered different from other skin squamous cell carcinoma. This is due to the invasiveness and predictive dissemination of specific risk factors of inguinal and pelvic lymph nodes. The majority will arise from the mucosal surfaces of the glans and inner prepuce. Most penile cancers are thought to develop through a series of changes in the squamous epithelium leading to penile intraepithelial neoplasia (PeIN). Human papilloma virus (HPV) is considered a high‐risk factor [6]. HPV‐associated PeIN leads to undifferentiated PeIN, while HPV‐independent PeIN is called differentiated PeIN and is likely driven by lichen sclerosus [7]. The Wnt signalling pathway, a key carcinogenic signalling network, has been implicated in many epithelial cancers in humans and is also reported to be deregulated in human penile SCC [1]. However, due to the rarity of the disease, detailed carcinogenic mechanisms are not clearly described yet.

The availability of human tissue and primary or immortal cell lines from human tissue constitute essential research tools for understanding detailed mechanisms of diseases and are necessary for developing and testing therapies. Due to the rarity of penile cancer, there is limited availability of penile cancer tissue samples and limited recruitment for trials investigating therapies. This scarcity has hindered the understanding of the cellular and molecular mechanisms of penile cancer. Few efforts have produced a limited number of penile cancer‐derived cell lines and none are currently commercially available [8, 9, 10, 11, 12, 13]. One study analysed two lines from a primary tumour and its lymph node metastases, reporting differential regulation of podoplanin and the chemokine CXCL14 [12]. A panel of five HPV‐negative penile cancer lines were generated from three lymph node metastases, from a recurrent local lesion or from a scrotal invasion lesion, with characterisation of their mutational burden and drug sensitivity [13] including in a HPV‐negative cell line [14]. Another group established three epithelial and three cancer‐associated fibroblast lines produced from verrucous or histologically usual penile SCC [10, 11]. They characterised the ability of these cells to form xenografts in immunocompromised mice [11] and also compared the penile cancer lines' mutation and gene expression profiles to foreskin cells from a healthy individual and assessed the sensitivity of the different lines to cisplatin and EGFR inhibitors [10]. Finally, a recent investigation generated two HPV‐positive penile cancer cell lines and reported HPV‐mediated upregulation of stem cell‐associated factors in the absence of Wnt pathway activation [9]. These studies and culture models have begun to provide useful insights into the biology of penile cancer but additional tools and systems are needed to improve our understanding of this disease.

Here, we report the generation of two new primary penile cancer cell lines and their detailed cellular and molecular characterisation. We have generated two primary cell lines from fresh penile cancer tissue samples from patients undergoing penile surgery to resect the tumour that were grown as matched normal and squamous cell carcinoma lines from the same patient tissue samples. We performed molecular karyotyping, gene and protein expression analyses for a comprehensive investigation of one paired cell line.

We further provide an interactome map of the genomic differences between paired normal and cancer cell lines. Tumour cell lines exhibited chromosomal aberrations as well as gene expression alterations when compared with their normal counterparts derived from the same patient.

## 2 Materials and methods

### 2.1 Tissue procurement and ethical approval

Samples from two patients undergoing surgery to remove penile cancer (sample 1 termed 2711, patient age 61 years; sample 2 termed 3107, patient age 71 years) were collected. Human tissue was collected with full and informed, written, patient consent and was approved and subject to institutional ethical review in accordance with the UCL Tissue Biobank (Research Ethics Committee reference 20/YH/0088). The study conforms to the ethical standards set by the Declaration of Helsinki. Tissue samples were obtained from surplus material sent for histological examination. No patient or clinical information or identification information, other than the age, was available to the experimenters. Both tissue samples were tested by clinical pathologists for HPV (by polymerase chain reaction and p16INK4A staining) and were found to be HPV positive. All methods were performed in accordance with the relevant guidelines and regulations. The surgeon identified the tumour and normal adjacent sample by visual examination during the procedure. A small piece (~1 mm^3^) of the normal and tumour tissue samples were then collected in separate sterile tubes with different instruments in order to prevent cross‐contamination. The samples were immediately transferred to the laboratory (within 15–20 min) where the tissue was processed for culturing.

### 2.2 Tissue culture and generation of primary lines

Once in the laboratory, all manipulations were performed in a Class II microbiological safety cabinet in a containment level 2 (CL2) laboratory. The normal and tumour tissue samples were sterilised by immersing in 10% v/v Povidone‐Iodine solution (Fisher Scientific, Waltham, MA, USA) for 10s, then washed quickly twice in 70% alcohol for 3 s and finally in Dulbecco's modified Eagle's medium (DMEM) with serum. A modification of the technique described by Rheinwald et al. [15] was used to prepare and propagate normal and cancer penile carcinoma cultures. Whenever possible, each tissue sample was dissected into two or more pieces before transferring with a Pasteur pipette to a T25 flask or tissue culture grade 60 mm diameter petri dish. Sterile 13 mm diameter glass coverslips were then placed individually over each piece of tissue to keep it in position, and a drop of growth medium was added to prevent drying. The petri dish was placed in an incubator for 24–48 h at 37 °C, then culture medium, 3 parts DMEM (high glucose 4.5 g·L^−1^; 5 mm l‐glutamine) and 1 part Ham's F12 containing 100 IU·mL^−1^ penicillin and 100 μg·mL^−1^ streptomycin then further supplemented with 1.8 × 10^−4^ m adenine, 0.5 μg·mL^−1^ hydrocortisone, 5 μg·mL^−1^ insulin, 10^−10^ m cholera toxin and 10 ng·mL^−1^ epidermal growth factor and 10% FCS was added dropwise to the flask. The tissue samples remained in the incubator at 37 °C, and the medium was changed twice a week. Microscopic examination of the edges of the tissue was performed daily to verify the presence of cellular outgrowths. When new SCC cells started to appear, a feeder layer of 6 × 10^5^ mitotically inactivated J2‐3T3 cells was added to the culture vessel. The SCC cells were allowed to grow out from each explant then passaged to a new flask containing a fresh feeder layer before confluence was reached, usually within one week. All experiments were performed using cells between passage three and five. Aliquots of 10^6^ cells were made in 10% dimethyl sulfoxide (DMSO)/bovine serum as soon as numbers allowed, then stored in liquid nitrogen for future use.

### 2.3 Immunocytochemistry

Immunocytochemistry was performed in two paired cell lines (N2711 and T2711 and N3107 and T3107). Normal and cancer lines were grown on sterile glass coverslips, washed in phosphate buffered saline (PBS) and fixed in fresh 4% formaldehyde for 10 min at RT. Fixed cells were washed twice with PBS, followed by permeabilisation with 0.1% Tween for 3 min, washed with PBS, blocked in 10% foetal calf serum (FCS), washed twice and incubated with the primary antibodies. The following monoclonal primary antibodies were used: HECD‐1 [16], anti‐E‐cadherin (E‐Cad, 1.1 mg·mL^−1^) Thermo Fisher (Waltham, MA, USA): 13–1700, at 1:100. P5D2 [12], anti‐integrin β1 (ITGB1, 1 mg·mL^−1^) DHSB, Iowa: AB‐528308, at 1:100. LH8 [17], anti‐keratin 5 (K5, 1 mg·mL^−1^) Abcam (Cambridge, UK): ab20203, at 1:100. LH2 [17], anti‐keratin 10 (K10, 1 mg·mL^−1^) Abcam (Cambridge, UK): ab20208, at 1:100. LL002 [18], anti‐keratin 14 (K14, 1.1 mg·mL^−1^) Thermo Fisher: MA5‐11599, at 1:100. LL0025 [19], anti‐keratin 16 (K16, 0.3 mg·mL^−1^) Thermo Fisher: MUB0351P, at 1:50. SY5 [20], anti‐involucrin (IVL, 1 mg·mL^−1^) Thermo Fisher: MA5‐11803, at 1:100. BC1 [21], anti‐transglutaminase 1 (TGM1, 1 mg·mL^−1^) at 1:100. BC1 antibody was a gift from Robert H. Rice (University of California, Department of Environmental Toxicology).

Cells were incubated with the primary antibody for 1 h at RT washed 3× for 2 min each. The secondary antibody, conjugated with Alexa Fluor 488 rabbit anti‐mouse (Thermo Fisher, 2 mg·mL^−1^) at 1:400, was added to the cells and incubated for 1 h at RT. Following the secondary antibody incubation, cells were washed in PBS and then in distilled H_2_O. Coverslips were mounted on a clean slide with a solution of Mowiol mounting medium (Calbiochem, Merck, Darmstadt, Germany) and 4′,6‐diamidino‐2‐phenylindole (DAPI) nuclear stain.

### 2.4 Fluorescence and confocal microscopy

For comparison across different samples and standardisation all parameters during image acquisition were kept constant throughout each experiment. For a global view of the staining of the whole coverslips, imaging was performed using an AxioScan Z1 (Carl Zeiss, Jena, Germany) multifluorophore slide scanner in conjunction with ZEN (Zeiss Microscopy, Munich, Germany) software. Confocal imaging of cells was also performed using a Leica TCS SP8 confocal microscope (Leica Microsystems, Wetzlar, Germany) with a 63× oil immersion objective lens (Numerical Aperture = 1.4) and the LASX Software (Leica Microsystems). The two fluorophores used were Alexa 488 and DAPI, excited, respectively, at 488 nm and 358 nm and their emission detected at, respectively, 519 nm and 461 nm. 1024 × 1024 Z‐stacks were collected with a Z‐step of 0.3 μm and optical sections of 0.894 μm; the maximum projections of these stacks were kept.

We also employed a quantitative, unbiased, automated particle analysis method to quantify the amount of expression of each antibody in the normal and tumour cells described elsewhere [22]. A Python script was written (Data S1) with the open‐source libraries ‘Python Imaging Library’, ‘glob’ and ‘operator’ to keep only the Alexa 488 channel (green colour), apply a threshold and count the amount of signal. Units were pixels; values were then standardised on the total amount of pixels on the images to give a quantitative immunofluorescence score. The significance of observed differences was verified with the Mann–Whitney U‐test on MedCalc (MedCalc Software). Box plots were constructed using OriginPro (OriginLab, Northampton, MA, USA).

### 2.5 Short tandem repeat (STR) analysis

The genomic DNA of the cells was isolated by using QIAamp DNA Mini Kit (Qiagen). PowerPlex assay (Promega, Madison, WI, USA) for the STR profiles of the cells were performed by Source BioScience (Nottingham, UK). The following loci were tested: AMEL, CSF1PO, D13S317, D16S539, D18S51, D21S11, D3S1358, D5S818, D7S820, D8S1179, FGA, Penta D, Penta E, TH01, TPOX, vWA. STR profiles of our cells were compared with other cell lines by STR Similarity Search Tool Cellosaurus 1.1.0 (ExPASy).

### 2.6 Molecular karyotyping

Trypsinised normal and cancer cell lines (N2711, N3107, T2711 and T3107) were used to isolate genomic DNA (PureLink Genomic DNA mini kit, Invitrogen, Waltham, MA, USA) according to the manufacturer's protocol. Eluted DNA samples were quantified using Nanodrop (Thermo Scientific). DNA (50 μg·mL^−1^) was used to carry out CytoScan HD (Affymetrix) independently by Atlas Biolabs (Berlin, Germany) using established protocols. Karyotyping analysis was conducted using Chromosome Analysis Suite (ChAS), version 4.3 (Thermo Fisher Scientific), where the CyScan HD raw files were uploaded to the software as per the manufacturer's instructions. ChAS was used to determine QC metrics for the CytoScan HD array (Affymetrix). Three different QC metrics were generated: 1. Median of the absolute values of all pairwise differences (MAPD), waviness standard deviation (SD) and single nucleotide polymorphism QC (SNPQC). Then, the differential gains and losses in all chromosomes option was used to identify the unique gains and losses per sample. Regions which showed overlapping between the two normal samples compared to the tumour ones were filtered.

### 2.7 RNA sequencing (RNA‐seq)

RNA was isolated using a Qiagen RNAeasy kit (Qiagen), and RNA‐seq was carried out on RNA extracted from N2711, N3107, T2711 and T3107 lines using targeted RNA‐seq. The library preparation was carried out using AmpliSeq Transcriptome (Thermo Fisher Scientific). A barcoded cDNA library was first generated using SuperScript VILO cDNA Synthesis kit from 20 ng of total RNA treated with Turbo DNase (Thermo Fisher Scientific), followed by amplification using the AmpliSeq transcriptomic technology. The quality control of the amplified cDNA libraries was analysed using Agilent Bioanalyzer high‐sensitivity chips. Libraries were then diluted to 100 pM and pooled equally, with two individual samples per pool. Pooled libraries were amplified using emulsion PCR with Ion Torrent OneTouch2 instruments (OT2) following the manufacturer's instructions and then sequenced using Ion Torrent Proton sequencing system, using Ion PI kit and chip version 2.

RNA‐seq data were analysed using the Ion Torrent Software Suite version 5.4. Alignment was carried out using the Torrent Mapping Alignment Program (TMAP). TMAP is optimised for Ion Torrent sequencing data to align the raw sequencing reads against the reference sequence derived from the hg19 (GRCh37) assembly. To maintain specificity and sensitivity, TMAP implements a two‐stage mapping approach. First, four alignment algorithms, BWA‐short (BWA, http://bio‐bwa.sourceforge.net), BWA‐long, SSAHA (sanger.ac.uk/tool/ssaha/), and Super‐maximal Exact Matching were employed to identify a list of candidate mapping locations. A further alignment process was performed using the Smith–Waterman algorithm [23] to find the final best mapping. Raw read counts of the targeted genes were performed using samtools (samtools view –c –F 4 –L bed_file bam_file). The quality control, including the number of expressed transcripts, was checked after Fragments Per Kilobase Million (FPKM) normalisation.

### 2.8 Identification of differentially expressed genes

Transcriptomic analysis was performed using the R/Bioconductor package NOISeq [24] with raw read counts. Read count normalisation was performed using NOISeq Genes with less than ten normalised read counts excluded from further analysis. Differentially expressed genes (DEG) determination was carried out using the NOISeq library as described previously [25], while the heatmap for the top DEGs and clustering was performed using the Pheatmap package.

### 2.9 Gene Ontology analysis and visualisation

Over and under‐expressed genes were subject to functional analysis using a hypergeometric algorithm based on Gene Ontology analysis using the Generally Applicable Gene‐set Enrichment for Pathway Analysis package (GAGE) and visualised using the pathview package, a toolset for pathway‐based data integration and visualisation.

### 2.10 Estimation of stromal and immune signatures in the cultured samples

TIMER (http://timer.cistrome.org), a comprehensive resource for systematical analysis of immune infiltrates across diverse cancer types, was used to deconvolute the raw RNA‐Seq profile of each sample to estimate the stromal versus cancer purity and immune cell infiltration.

### 2.11 DEGs' chromosomal relation to chromosomal regions

The bioconductor package, karyoploteR, was used to create karyotype plots of arbitrary genomes and offers a complete set of functions to plot arbitrary data on them. DEGs were plotted on the regions that showed shared alternation in both cancer cell lines compared to the healthy ones.

### 2.12 RNA isolation and qPCR

The expression of E‐Cadherin, keratin 14, transglutaminase 1, integrin β1, keratin 5, keratin 10, keratin 16 and involucrin in the paired primary cells was assessed by quantitative real‐time PCR. Total RNA was extracted for cell lines using the Qiashredder and Qiagen RNeasy Mini kits (Qiagen) according to the manufacturer's instructions. The quality of the RNA was checked on a NanoDrop 2000 (Thermo Fischer Scientific). According to the manufacturer's protocol, cDNA was obtained after the mRNA's reverse transcription (RT) with the Omniscript RT Kit (Qiagen). PCR primers (Data S2) were designed from cDNA sequences of Ecad, TGM1, ITGB1, IVL, K5, K10, K14 and K16 using Primer3. Primers were designed to be 400 to 600 bp long. Statistical difference was evaluated with *t*‐tests.

### 2.13 RNAscope single‐molecule RNA detection

RNAscope single‐molecule RNA assay was procured from Advanced Cell Diagnostics Bio (ACDbio, Biotechne, Abingdon, UK). The probes were chosen to identify 16 high‐risk and 10 low‐risk HPV types (HPV‐HR18, 312 598, ACD and HPV‐LR10, 314 558, ACD, respectively). The RNAscope technique detects a wide range of, transcriptionally active, HPV types. Cells were grown on 3‐well chamber slides (Thistle Scientific Ltd, Rugby, UK cat. No. IB‐80381) as described earlier. All other procedures were similar to those described elsewhere [26]; negative (dapB) and Hs‐UBC controls were used as experimental controls as per the manufacturer's protocol. The sample was imaged using a fluorescence microscope (Olympus) and imported into imagej software [27].

## 3 Results

### 3.1 Establishment of primary cell lines from normal and tumour penile tissue samples

Of the twelve patient samples received, six contained both normal and tumour tissue; of these, two were contaminated and discarded. Two paired cell lines, termed 2711 and 3107, were successfully grown. Of the other two samples, only normal cultures grew without the corresponding carcinoma cell growth and were not considered further. Each pair of normal and tumour cell lines from the same patient was prefixed with N, and T. We performed detailed molecular and immunochemical characterisation of the paired cell lines derived from normal and tumour samples.

### 3.2 Cells derived from tumour tissue showed unique microscopic and macroscopic features

**Fig. 1:**
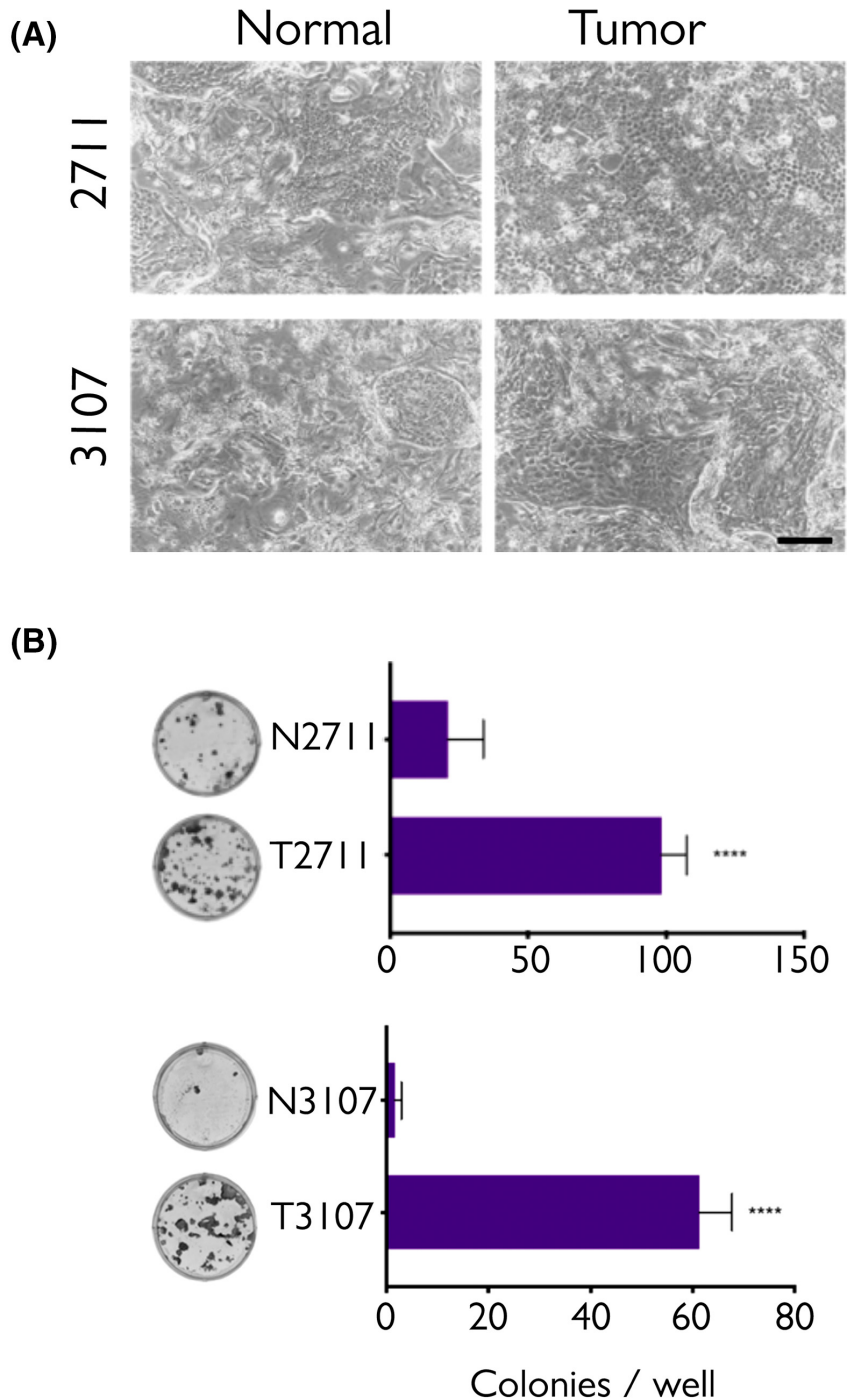
Morphology and functional characterisation of normal and tumour human penile keratinocytes. (A) Brightfield images of normal and tumour keratinocytes were isolated from two different individuals (2711 and 3107); the cells were monitored twice weekly during growth and images shown are representative of at least ten such observations (scale bar = 200 μm). Keratinocytes were grown atop a layer of mitotically inactivated fibroblasts. (B) Colony formation ability of normal (N) and tumour (T) keratinocytes. Shown are representative wells and quantification by manual counting of colonies in two hexaplicate experiments (*n* = 6, mean ± SEM **** = Student's *t*‐test *P* < 0.0001).

Cells derived from tumour tissue were small (5 μm), cobblestone in appearance and undifferentiated (Fig. 1A). Cells from normal samples were largely undifferentiated but contained large, differentiated cells in the middle of the colonies (Fig. 1A) and appeared closely bound compared to those from the tumour samples. Compared to normal (N), tumour (T) keratinocytes showed more than 10‐fold increase in colony formation ability (Fig. 1B).

The STR profiles of the tumour and healthy tissue cells were identical, and there were no matching STR profiles among commonly contaminating cell lines (Data S3).

### 3.3 Normal and tumour‐derived cell lines showed differential expression of basal cells or differentiated layers in the squamous epithelium markers

**Fig. 2:**
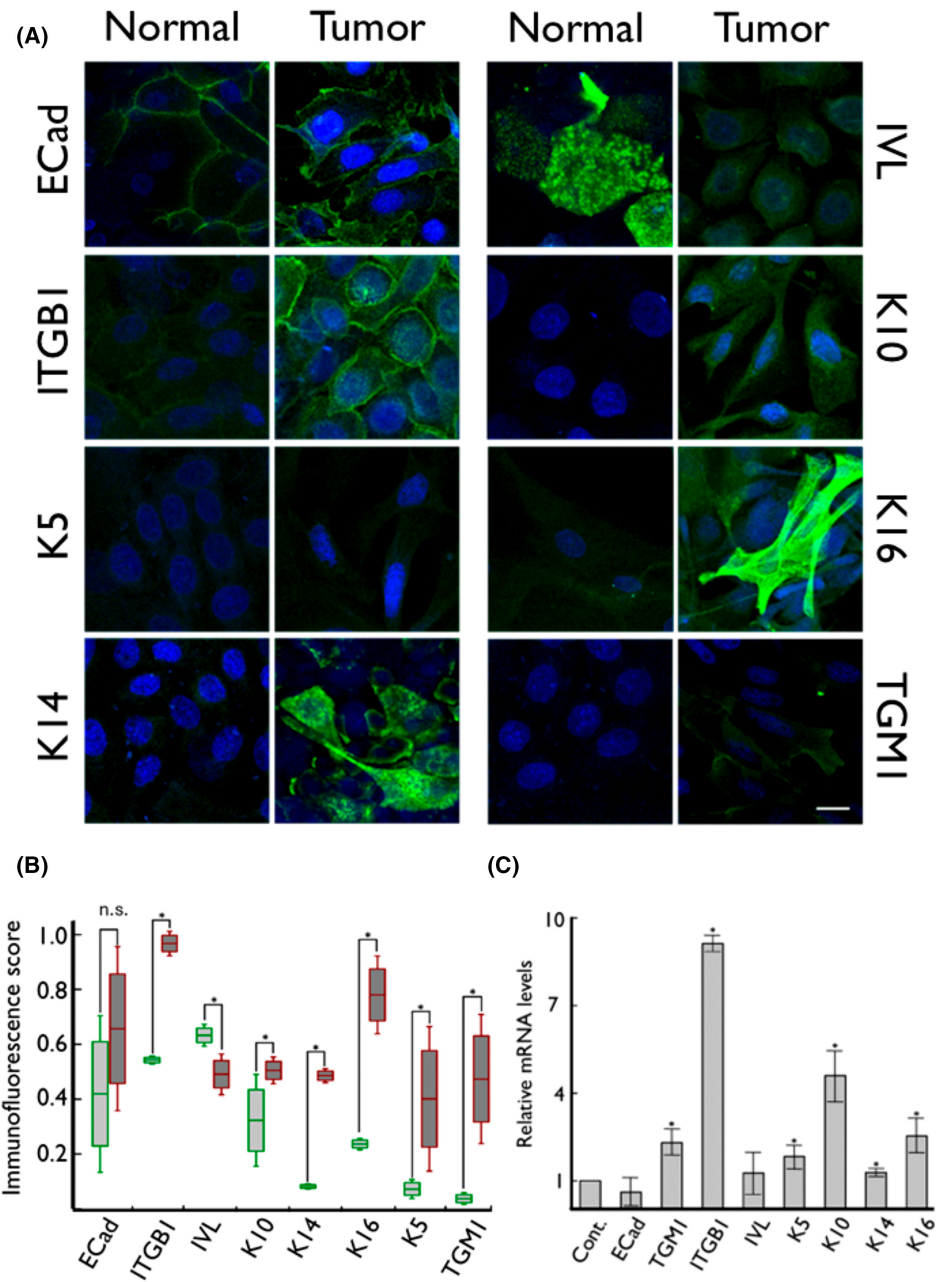
Expression of epidermal markers in normal and tumour human penile keratinocytes. (A) Immunofluorescent staining of normal and tumour penile keratinocytes (*N* = 3). Representative fields show merged confocal imaging of nuclei (DAPI, blue) and a panel of epidermal marker proteins (green). The scale bar is 10 μm. (B) Quantification of the immunofluorescence images was performed using a Python script. For details on the Quantitative Immunofluorescence Score, see Materials and Methods. Data are presented as box plots; green boxes represent normal cells; red boxes represent tumour cells (immunofluorescence experiments were performed in 3 independent passages; * = Mann–Whitney U‐test *P* < 0.05; n.s. = Mann–Whitney U‐test *P* > 0.05). (C) Changes in epidermal marker mRNA levels in tumour cells compared to matched normal cells relative to GAPDH housekeeping gene expression (*n* = 3, mean ± SEM, Student *t*‐test was used to measure significance).

We used a panel of antibodies, including E‐cadherin (ECad) (general epithelial marker) [28], integrin β1 (ITGB1) [29], keratin 14 (K14), keratin 5 (K5, basal markers), tissue transglutaminase 1 (TGM1) [30], keratin 16 (K16, hyperproliferation marker) keratin 10 (K10) and involucrin (IVL, differentiation markers), to detect a number of proteins known to be markers of basal cells or differentiated layers in the squamous epithelium (Fig. 2).

ECad, a transmembrane glycoprotein, is a member of the superfamily of cadherins [28]; ECad was widely expressed in both normal and tumour‐derived cells (Fig. 2A) without a significant difference between normal epithelial and tumour cells when quantified (Fig. 2B). However, tumour cells showed significantly higher expression for the basal markers ITGß1, K5 and K14. ITGß1 is a subunit of the integrin family of proteins that form a major class of cell adhesion proteins [29] ITGß1 was increased in the tumour samples compared to normal, an observation confirmed through quantitation (Fig. 2B). Keratin proteins are part of a large family and constitute the vast majority of the intermediate filaments of the cytoskeleton of epithelial cells [31]. K5 and K14 are expressed in keratinocytes of stratified squamous epithelium and are characteristic features of squamous cell carcinoma [32]. K16 is also expressed in some squamous epitheliua and linked to an altered differentiation process related to hyperproliferation [33]. The expression of K5, K10, K14 and K16 was increased in tumours compared to normal cells (Fig. 2A,B). In the case of K5, the increase amounted to nearly 5‐fold. A number of cells positive for differentiation markers K10, IVL and TGM1 can be detected among normal and tumour cells. IVL is a cytoplasmic protein expressed in keratinocytes that have left the basal layer, and it is used as a marker of the terminal differentiation of squamous epithelial cells [34]. Transglutaminases are proteins involved in the formation of the cornified layer by catalysing the cross‐linking of different structural proteins during epidermal differentiation [30]. The expression of IVL was downregulated in tumour cells, whereas K10 and TGM1 were upregulated (Fig. 2B). Interestingly, quantitative analysis showed a significant, nearly 10‐fold increase in the basal level of TGM1 expression in tumours compared to normal cells (Fig. 2A,B). ITGB1, K5, K14, K10, K16 and TGM1 also displayed increased expression in tumour cells at the mRNA level (Fig. 2C).

### 3.4 Unique multiple aberrations in the DNA derived from the tumour cell

**Fig. 3:**
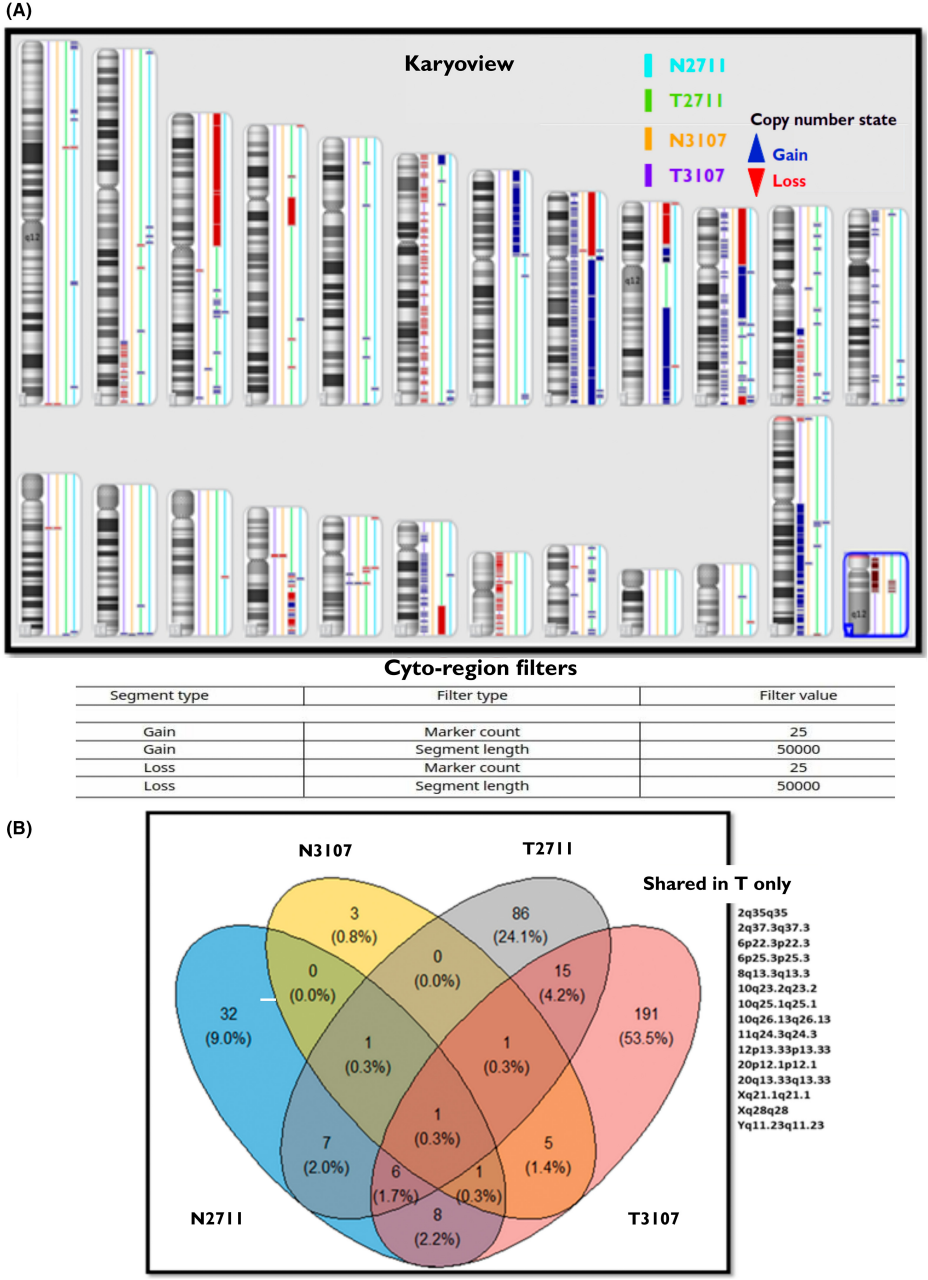
Molecular karyotyping of the two paired cell lines (N2711, T2711 and N3107, T3107) was performed using an Affymetrix CytoScan HD chip. The Figure shows a ‘Karyoview’ diagram of N2711, T2711 and N3107 and T3107 cell lines of all detected genome‐wide copy number variants (losses highlighted in red) greater than or equal to 25 markers and gains (highlighted in blue) greater than or equal to 50 markers. Multiple aberrations in the DNA derived from the tumour cell line are highlighted as shared in T, involving 15 regions in chromosomes 2, 6, 8, 10, 11, 12, 20, X and Y.

A genomic screen was performed in two paired cell lines using the CytoScan HD array shown in the ‘Karyoview’ (Fig. 3A). Tumour cell lines had 42 gains and 11 losses in the patient 2711, while the patient 3107 tumour cell line had 14 gains and 22 losses. Fifteen genomic aberrations were specific to the tumour cell lines in both patients, involving loci on chromosomes 2, 6, 8, 10, 11, 12, 20, X and Y (Fig. 3B). Out of these 15 aberrations, only eight cytobands were identical in both (8q13.3, 10q23.2, 10q25.1, 10q26.13, 12p13.33, 20q13.33, Xq21.1) or lost in both (Yq11.23) as listed in Table 1. There are limited investigations of karyotyping of penile cell lines and tissue samples. These are a heterogenous and distinct set of gains and losses compared to previously described investigations [35, 36, 37], indicative of population heterogeneity and that seen in cancer, generally.

**Table 1:** Cytoband alterations in common in both tumour samples. Gain and loss of cytobands common in both tumour samples compared to normal. A vast majority of alterations are typified by gains and on chromosome 10 followed by 20. A total of 93 genes appear to be affected primarily. CN, copy number; Marker count, number of CN variation. Red=gain, Blue=loss, light blue=T3107, light red=T2711.

File	CN state	Type	Chromosome	Cytoband start	Cytoband end		Size (kbp)	Marker count	Gene count	Genes
T2711	3	Gain	8	q13.3	q13.3	8q13.3q13.3	491.67	480	1	EYA1
T3107	3	Gain	8	q13.3	q13.3	8q13.3q13.3	66.84	186	1	EYA1
T2711	3	Gain	10	q23.2	q23.2	10q23.2q23.2	145.87	200	4	AGAP11, FAM25A, GLUD1, SHLD2
T3107	3	Gain	10	q23.2	q23.2	10q23.2q23.2	186.87	380	2	GRID1, MIR346
T3107	3	Gain	10	q23.2	q23.2	10q23.2q23.2	95.65	83	1	WAPL
T3107	3	Gain	10	q23.2	q23.2	10q23.2q23.2	76.15	72	1	SHLD2
T2711	3	Gain	10	q25.1	q25.1	10q25.1q25.1	90.30	127	1	SORCS3
T3107	3	Gain	10	q25.1	q25.1	10q25.1q25.1	169.42	228	7	CFAP43, MIR609, GSTO1, MIR4482, GSTO2, ITPRIP, ITPRIP‐AS1
T3107	3	Gain	10	q25.1	q25.1	10q25.1q25.1	78.16	108	1	SORCS3
T3107	3	Gain	10	q25.1	q25.1	10q25.1q25.1	289.42	212	2	XPNPEP1, ADD3‐AS1
T2711	3	Gain	10	q26.13	q26.13	10q26.13q26.13	74.51	108	2	TACC2, BTBD16
T2711	3	Gain	10	q26.13	q26.13	10q26.13q26.13	349.00	216	1	CHST15
T2711	3	Gain	10	q26.13	q26.13	10q26.13q26.13	101.77	130	2	FAM53B, FAM53B‐AS1
T3107	3	Gain	10	q26.13	q26.13	10q26.13q26.13	277.50	346	5	BTBD16, PLEKHA1, MIR3941, ARMS2, HTRA1
T3107	3	Gain	10	q26.13	q26.13	10q26.13q26.13	145.18	180	4	DMBT1, LOC112577516, C10orf120, DMBT1L1
T2711	3	Gain	12	p13.33	p13.33	12p13.33p13.33	253.08	254	3	TULP3, TEAD4, TSPAN9
T3107	3	Gain	12	p13.33	p13.33	12p13.33p13.33	52.91	116	1	ERC1
T3107	3	Gain	12	p13.33	p13.33	12p13.33p13.33	107.31	78	2	TULP3, TEAD4
T2711	3	Gain	20	q13.33	q13.33	20q13.33q13.33	328.43	340	5	CDH26, C20orf197, LOC729296, MIR646HG, MIR646
T2711	3	Gain	20	q13.33	q13.33	20q13.33q13.33	136.42	148	7	LSM14B, PSMA7, SS18L1, MTG2, HRH3, LOC105369209, OSBPL2
T2711	3	Gain	20	q13.33	q13.33	20q13.33q13.33	104.16	84	2	NTSR1, LINC00659
T3107	3	Gain	20	q13.33	q13.33	20q13.33q13.33	133.21	136	6	PSMA7, SS18L1, MTG2, HRH3, LOC105369209, OSBPL2
T2711	2	Gain	X	q21.1	q21.1	Xq21.1q21.1	177.26	57	0	
T3107	2	Gain	X	q21.1	q21.1	Xq21.1q21.1	5638.60	4144	20	ATRX, MAGT1, COX7B, ATP7A, PGAM4, PGK1, TAF9B, CYSLTR1, RTL3, LPAR4, MIR4328, P2RY10, GPR174, ITM2A, TBX22, CHMP1B2P, TENT5D, BRWD3, HMGN5, SH3BGRL
T2711	0	Loss	Y	q11.23	q11.23	Yq11.23q11.23	161.69	236	0	
T3107	0	Loss	Y	q11.23	q11.23	Yq11.23q11.23	1039.36	232	12	TTTY17A, TTTY17B, TTTY17C, TTTY4, TTTY4B, TTTY4C, BPY2, BPY2B, BPY2C, DAZ3, DAZ4, DAZ2
T3107	0	Loss	Y	q11.23	q11.23	Yq11.23q11.23	1029.68	776	4	CDY1, CDY1B, TTTY3, TTTY3B

Notably, several of these loci (e.g. 10q23.2–10q26.13 and 20q13.33) harbour genes involved in cell cycle regulation, DNA repair and oncogenic signalling, but have not previously been reported as recurrent sites of alteration in penile squamous cell carcinoma (PSCC). This suggests that our cell lines capture novel, population‐specific and tumour‐intrinsic genomic events that extend beyond the canonical alterations described in the literature. The divergence from previously published karyotyping studies underscores the genomic heterogeneity of PSCC and highlights the value of these paired cell lines as unique resources for dissecting the functional consequences of chromosomal instability in penile cancer.

### 3.5 Differentially expressed genes in tumour cells

**Fig. 4:**
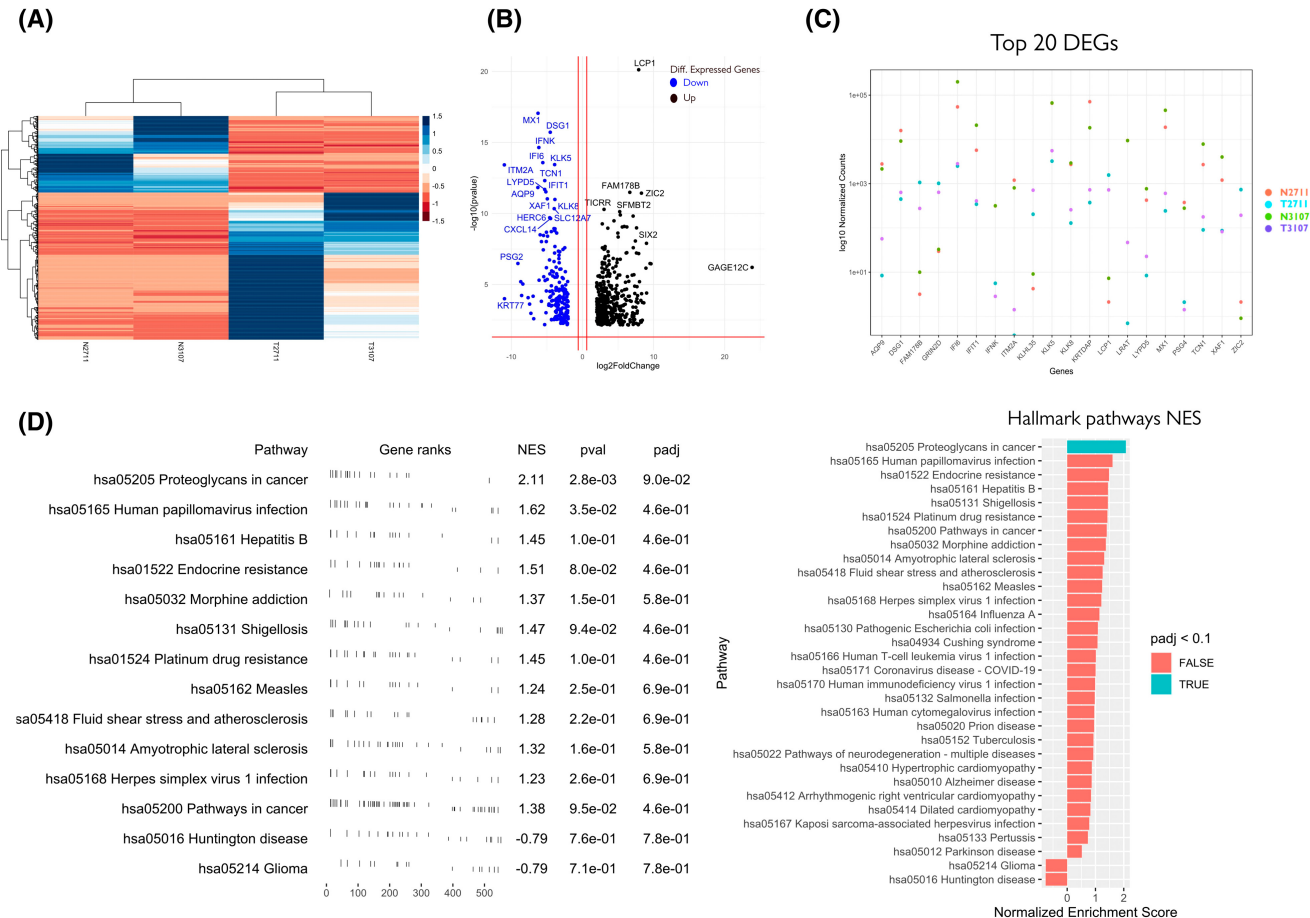
Transcriptomic profiling for two paired cell lines (four individual samples from N2711, T2711 and N3107, T3107) using RNA‐Seq. (A) Heatmap of the top differentially expressed genes (DEGs). (B) Volcano plot showing DEGs with log_2_fold > 2 or <2, adj*P* < 0.05. The log*P* in a NOISeq volcano plot comes from the NOISeq probability score (*q*‐value) that reflects the likelihood of differential expression, transformed into log scale (usually −log_10_) for visualisation. (C) Normalised gene expression of the top 20 DEGs (D) Top pathways in which the top DEGs are enriched. Other pathways such as Human papillomavirus infection (hsa05165) are shown. DEGs that showed significant upregulation in tumour are highlighted red, while those downregulated were highlighted with green.

RNA‐seq was performed to understand the global transcriptomic changes specific to tumour cell lines compared to their healthy counterparts: 644 DEGs were identified, as shown in Fig. 4A,B.

A graphical representation of normalised expression of top 20 DEGs is shown in Fig. 4C. These DEGs were enriched in disease‐related pathways, one of these is the proteoglycans in cancer (hsa05205), as shown in Fig. 4D. Proteoglycans are characterised by a central protein backbone decorated with various linear sulfated glycosaminoglycan side chains [38]. According to KEGG's Proteoglycans in cancer reference pathway (Table 2), there are four main types of proteoglycans, including hyaluronan (HA), which does not occur as a PG but in free form, heparan sulfate proteoglycans (HSPGs), chondroitin sulfate proteoglycans (CSPGs), dermatan sulfate proteoglycans (DSPG) and keratan sulfate proteoglycans.

**Table 2:** Genes enriched in proteoglycans pathway. Differentially expressed genes (DEGs) enriched in pathways related to proteoglycans in cancer (hsa05205) as interrogated using the Kyoto Encyclopedia of Genes and Genomes (KEGG) is a bioinformatics resource for deciphering the genome and can be used to analysed DEGs in the pathview. The table shows DEGs that showed significant upregulation in tumour compared to normal using the KEGG pathway analysis. There are four key proteoglycan pathways in the KEGG Key: CSPG, Chondroitin sulfate proteoglycans; HA, Hyaluronan; HSPG, Heparan sulfate proteoglycans; KSPG, Keratan sulfate proteoglycan.

HA	HSPGs	CSPG	KSPG
Raf‐1	Raf‐1	AKT	FasL
Cyclin D1	AKT	Casp3	Fas
F‐Actin	Actin		
Ankyrin			
AKT			
RhoA			
CAMKII			

### 3.6 The top 20 DEGs are involved in immune responses like interferon alpha/beta signalling

**Fig. 5:**
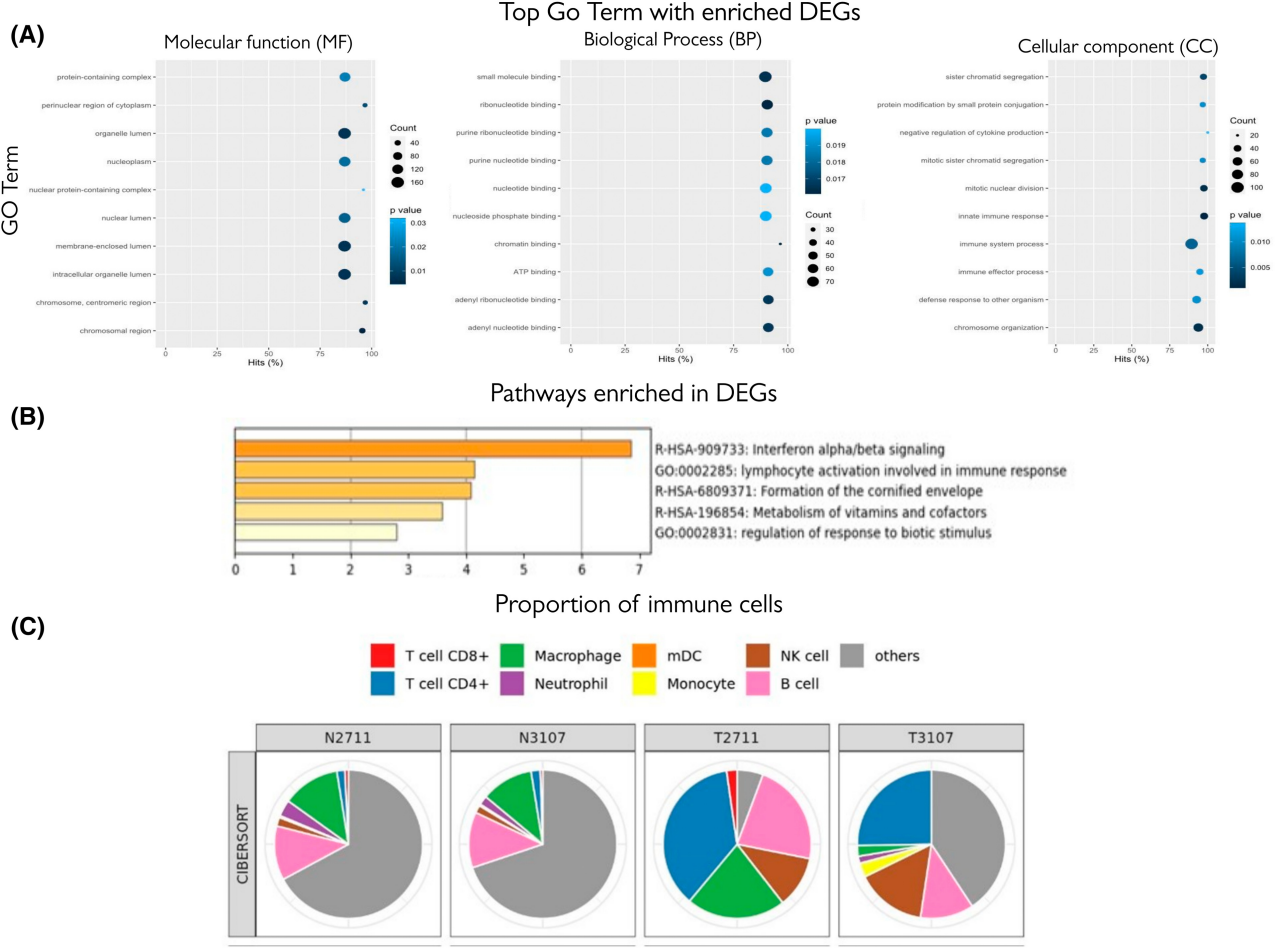
(A) Top Go Terms where differentially expressed genes (DEGs) are enriched significantly showing three categories of terms: Biological process (BP), cellular component (CC) and molecular function (MF). (B) The lower graph shows the top pathways enriched using the top 20 DEGs. Both immune‐related pathways showed significant enrichment. (C) The proportion of immune cells in each sample using TIMER to estimate the significance of the immune cell from the raw RNA‐Seq profile.

We compared our data to those in other available databases. DEGs in tumour samples showed enrichment in Gene Ontology (GO) (Fig. 5) Terms Biological process related to immune response (Fig. 5A) and a list of the top 20 DEGs is given in Table 3. For example, the top 20 DEGs (LCP1, MX1, DSG1, GRIN2D, KRTDAP, IFNK, PSG4, IFI6, ITM2A, KLK5, TCN1, AQP9, LYPD5, IFIT1, FAM178B, ZIC2, LRAT, XAF1, KLK8 and NNMT) (Fig. 5B) showed enrichment in pathways related to an immune response. Pathways such as those for interferon alpha/beta signalling (IFI6, IFIT1, MX1, XAF1, IFNK, LCP1, LRAT), lymphocyte activation involved in immune response (LCP1, ITM2A, IFNK) and formation of the cornified envelope (DSG1, KLK8, KLK5) showed the top enrichment as shown in Fig. 5A,B. Immune deconvolution of penile cancer‐derived cell lines further revealed an apparent enrichment of CD4^+^ T‐cell‐ and NK cell‐associated signatures in tumour lines (Fig. 5C). Notably, N3107 and T3107 shared a large undefined (‘other’) component, which was also prominent in T2711, suggesting common transcriptional patterns not captured by canonical immune categories. In contrast, the macrophage‐associated signature appeared reduced in T3107 compared with N2711, N3107 and T2711. Importantly, as these data were derived from cell lines rather than tissue, the inferred immune signatures are more likely to represent immune‐like transcriptional programs intrinsic to tumour cells than infiltration by actual immune subsets.

**Table 3:** Top 20 differentially expressed genes (DEGs) between tumour (T) and normal (N). A list of 20 most differentially expressed between T and N (>2 fold and an adjusted *P* value of <0.05) genes is shown.

Top 20 DEGs between T and N, log_2_ fold change >2 and adj *P* < 0.05, positive log_2_fold = more in T						
	baseMean	Log_2_foldchange	lfcSE	Stat	*P* value	*P* adj
LCP1	573.96	7.90	0.84	9.36	7.54E‐21	1.14E‐16
MX1	16292.54	−6.25	0.72	−8.58	8.76E‐18	6.64E‐14
DSG1	6523.65	−4.53	0.55	−8.22	1.90E‐16	8.70E‐13
GRIN2D	428.99	4.72	0.57	8.205	2.30E‐16	8.70E‐13
KRTDAP	22627.23	−6.34	0.79	−8.00	1.15E‐15	3.48E‐12
IFNK	159.70	−6.13	0.77	−7.92	2.25E‐15	5.67E‐12
PSG4	164.50	−7.46	0.96	−7.71	1.21E‐14	2.61E‐11
IFI6	64891.05	−5.57	0.73	−7.61	2.62E‐14	4.96E‐11
ITM2A	500.05	−10.98	1.45	−7.56	3.77E‐14	5.71E‐11
KLK5	35299.70	−3.91	0.51	−7.57	3.62E‐14	5.71E‐11
TCN1	2700.72	−5.30	0.73	−7.22	4.84E‐13	6.67E‐10
AQP9	1250.55	−6.27	0.88	−7.07	1.48E‐12	1.86E‐09
LYPD5	305.27	−5.31	0.75	−7.03	2.03E‐12	2.36E‐09
IFIT1	6843.78	−5.14	0.73	−6.97	2.98E‐12	3.22E‐09
FAM178B	338.26	6.65	0.95	6.96	3.23E‐12	3.26E‐09
ZIC2	232.44	8.29	1.19	6.94	3.70E‐12	3.51E‐09
LRAT	4726.27	−8.65	1.24	−6.93	3.99E‐12	3.56E‐09
XAF1	1343.45	−4.94	0.72	−6.81	9.33E‐12	7.85E‐09
KLK8	1506.64	−3.87	0.5	−6.80	1.03E‐11	8.23E‐09
NNMT	711.11	6.87	1.03	6.65	2.81E‐11	2.13E‐08

### 3.7 Eleven identified DEGs are frequently differentially expressed between PSCC, and normal adjacent tissue with and without metastasis

**Fig. 6:**
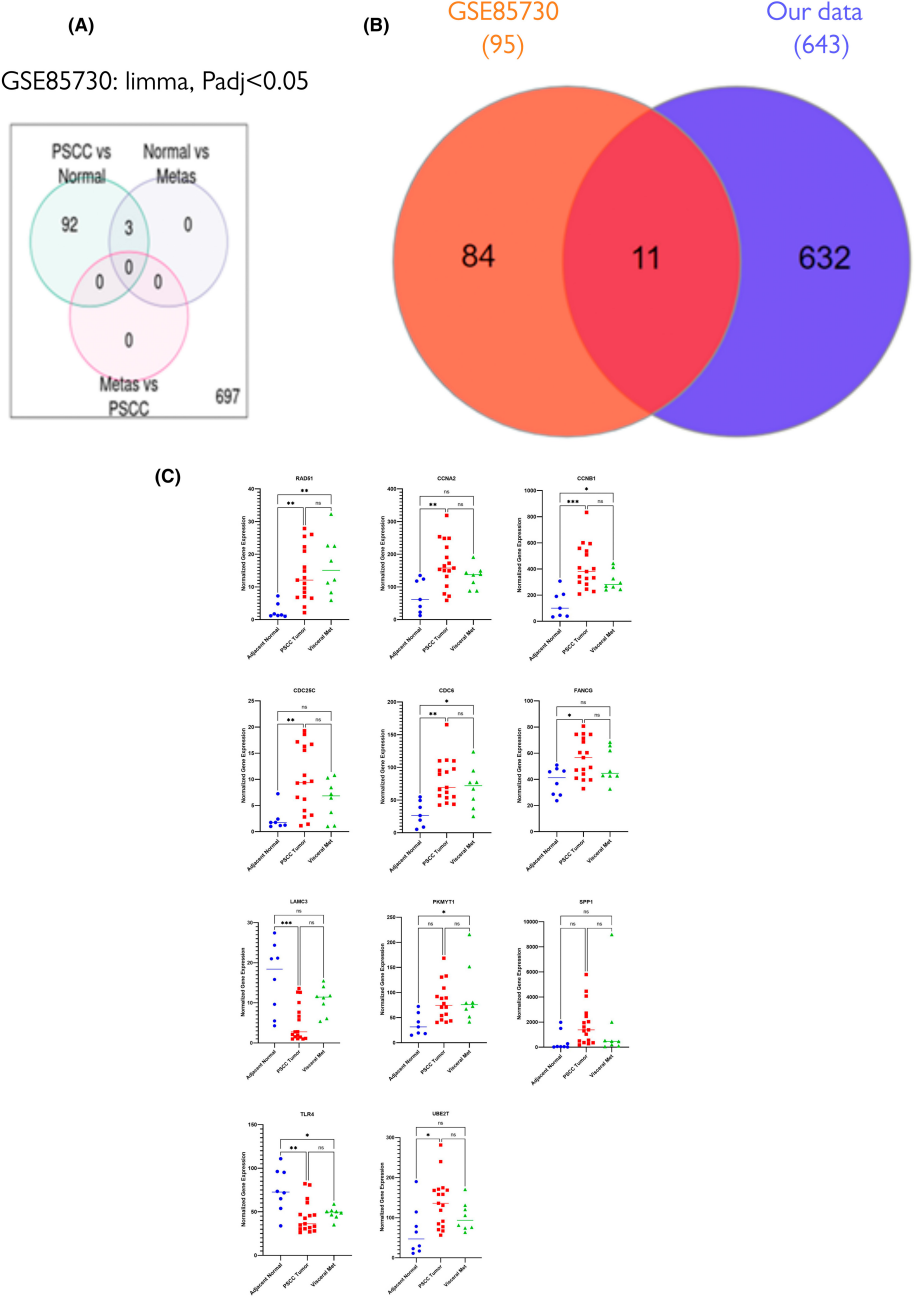
(A) Intersection between DEGs in our samples and those from the GSE85730 dataset of 25 patients (adjacent normal, *n* = 8; tumour without metastasis, *n* = 17; PSCC tumour with visceral metastasis, *n* = 8) with locally advanced or metastatic penile squamous cell carcinoma (PSCC). (B) Normalised gene expression of 11 DEGs shared between GSE85730 and our data (RAD51, CCNA2, CCNB1, CDC25C, CDC6, FANCG, LAMC3, PKMYT1, SPP1, TLR4 and UBE2T) is plotted. 8q13.3 and Xq21.1 cytobands gains are positively correlated with the fold change in expression of nearby genes. (C) Dot plots of the 11 DEGs from (B); horizontal lines indicate mean normalised gene expression in three conditions (adjacent normal, PSCC tumour without metastasis and PSCC tumour with visceral metastasis); ANOVA (analysis of variance) was used to test whether the means of the groups are significantly different.

We next compared our identified DEGs with the GSE85730 dataset of 25 patients with locally advanced or metastatic PSCC. Interestingly 11 DEGs were shared between GSE85730 and our data (RAD51, CCNA2, CCNB1, CDC25C, CDC6, FANCG, LAMC3, PKMYT1, SPP1, TLR4 and UBE2T). Normalised gene expression of each was extracted and plotted (Fig. 6). Analysis of the GSE85730 dataset (limma, *P* adj < 0.05) revealed 92 DEGs between penile squamous cell carcinoma (PSCC) and normal samples, with three genes shared between PSCC vs normal and normal vs metastatic comparisons, while no significant DEGs were identified when comparing metastases to PSCC (Fig. 6A). When we compared our dataset (643 DEGs) to GSE85730 (95 DEGs), we identified 11 overlapping genes that were consistently dysregulated across both datasets (RAD51, CCNA2, CCNB1, CDC25C, CDC6, FANCG, LAMC3, PKMYT1, SPP1, TLR4 and UBE2T) (Fig. 6B).

To assess the biological relevance of the 11 overlapping DEGs identified between our dataset and the public cohort GSE85730, we examined their normalised expression values across adjacent normal, PSCC tumour and visceral metastasis samples (Fig. 6C). Several genes associated with DNA damage repair and cell cycle regulation, including RAD51, CCNA2, CCNB1, CDC25C, CDC6 and FANCG, were significantly upregulated in PSCC tumours compared with adjacent normal tissues. This supports a transcriptional program consistent with enhanced proliferative and genomic instability pathways in penile carcinogenesis. Notably, their expression levels did not significantly differ between primary tumours and metastatic lesions, suggesting these alterations are established early during tumorigenesis and sustained throughout progression.

Genes linked to extracellular matrix remodelling and tumour aggressiveness, such as LAMC3, PKMYT1 and SPP1, were also elevated in tumours relative to normal samples, although no significant differences were observed between tumour and metastasis. Immune‐ and stress‐response genes showed mixed patterns: TLR4 expression was significantly reduced in tumours relative to normal samples, whereas UBE2T expression was increased, further pointing to immune‐modulatory alterations during tumour initiation.

Collectively, these findings confirm that the 11 overlapping DEGs represent functionally relevant alterations in PSCC. They highlight key biological pathways, for example DNA repair, cell cycle progression, extracellular matrix (ECM) remodelling and immune modulation, that distinguish tumour tissue from normal epithelium, while remaining largely stable across metastatic sites.

Although the majority of DEGs were unique to our dataset (*n* = 632), the shared subset provides independent support for the robustness of our findings. Notably, these overlapping genes include several immune‐ and stress‐response‐related transcripts, consistent with our earlier observation of immune pathway enrichment (e.g. Fig. 5). The relatively larger number of DEGs detected in our dataset likely reflects differences in experimental design, as our analysis was performed on cell lines that may capture additional tumour‐intrinsic transcriptional programmes not observed in bulk tissue analyses.

Taken together, these findings indicate that our data recapitulate key transcriptional features of penile cancer identified in independent cohorts, while also uncovering a broader immune‐like gene expression signature unique to our model system. This highlights both the relevance of our data set for comparative analysis putative insights into the molecular underpinnings of penile cancer biology.

### 3.8 EYA1 in 8q13.3 was significantly upregulated in both tumour samples

**Fig. 7:**
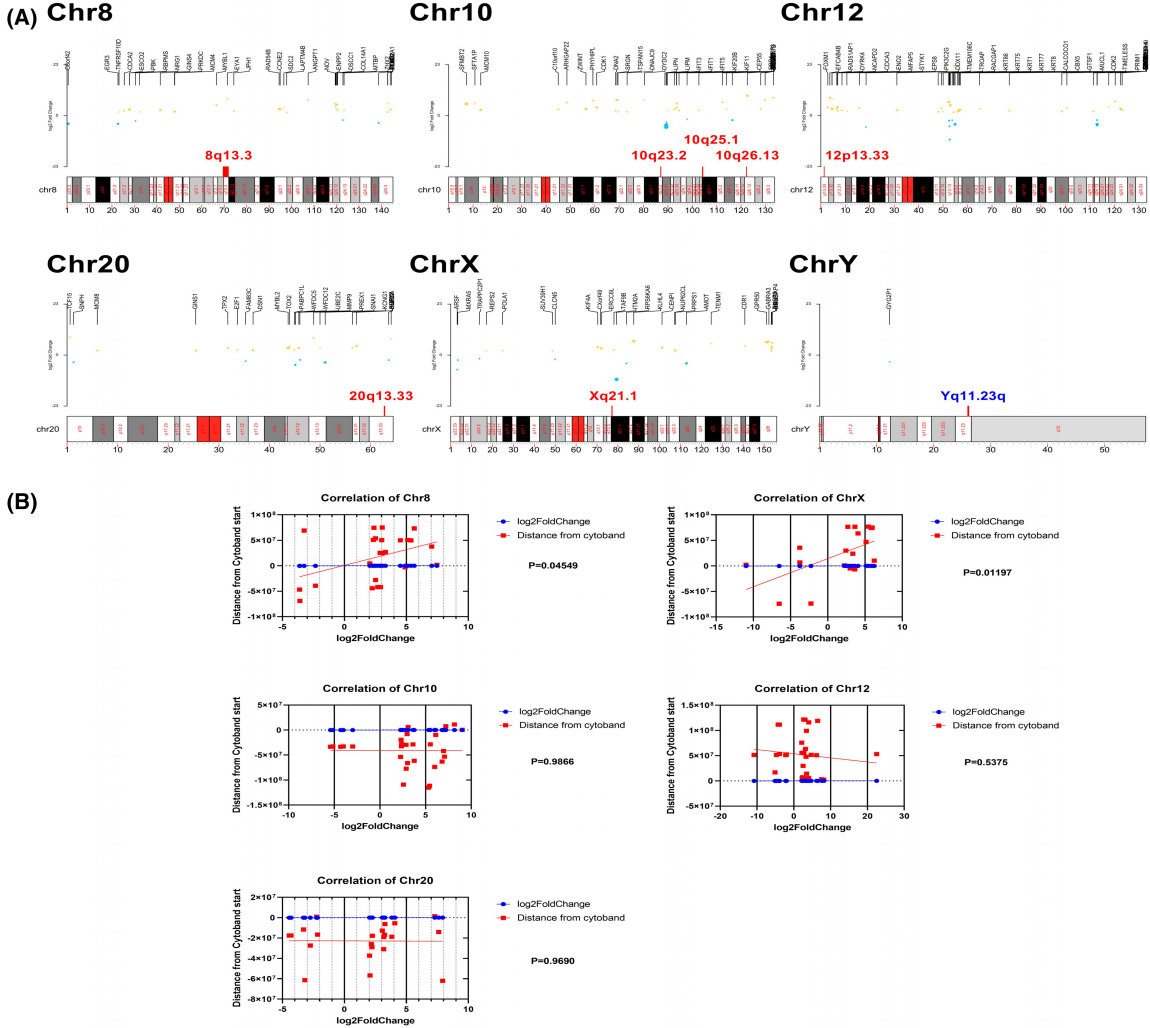
Differentially expressed genes plotted against karyotypes of the eight frequently altered cytobands in tumour cell lines. (A) Karyotype and DEG plots (B) linear correlation between differentially expressed genes (DEGs) start in each chromosome and the start of the cytoband altered in the given chromosome.

To understand the impact of the identified chromosomal aberrations on nearby gene expression, we mapped the significantly differentially expressed genes (DEGs) across each chromosome, highlighting the eight cytoband alterations that were common between the two tumour cell lines compared with their normal counterparts (Fig. 7A). We further examined the correlation between the genomic start position of each altered cytoband and the transcription start sites of the significantly altered genes (Fig. 7B). Interestingly, 8q13.3 and Xq21.1 cytobands gains are positively correlated with the fold change in the expression of nearby genes. A correlation between DEGs and karyotypes is shown in Table 4.

**Table 4:** Correlation between karyotypes and differentially expressed genes DEGs. Correlation between significantly differentially expressed genes plotted against karyotypes. See also Fig. 8.

Chromosomes	Sig DEGs	Correlation between cytoband start and sig DEGs log_2_Fold
chr8	24	0.41
chrX	19	0.56
chr20	22	ns
chr10	28	ns
chr12	32	ns
chrY	1	ns

**Fig. 8:**
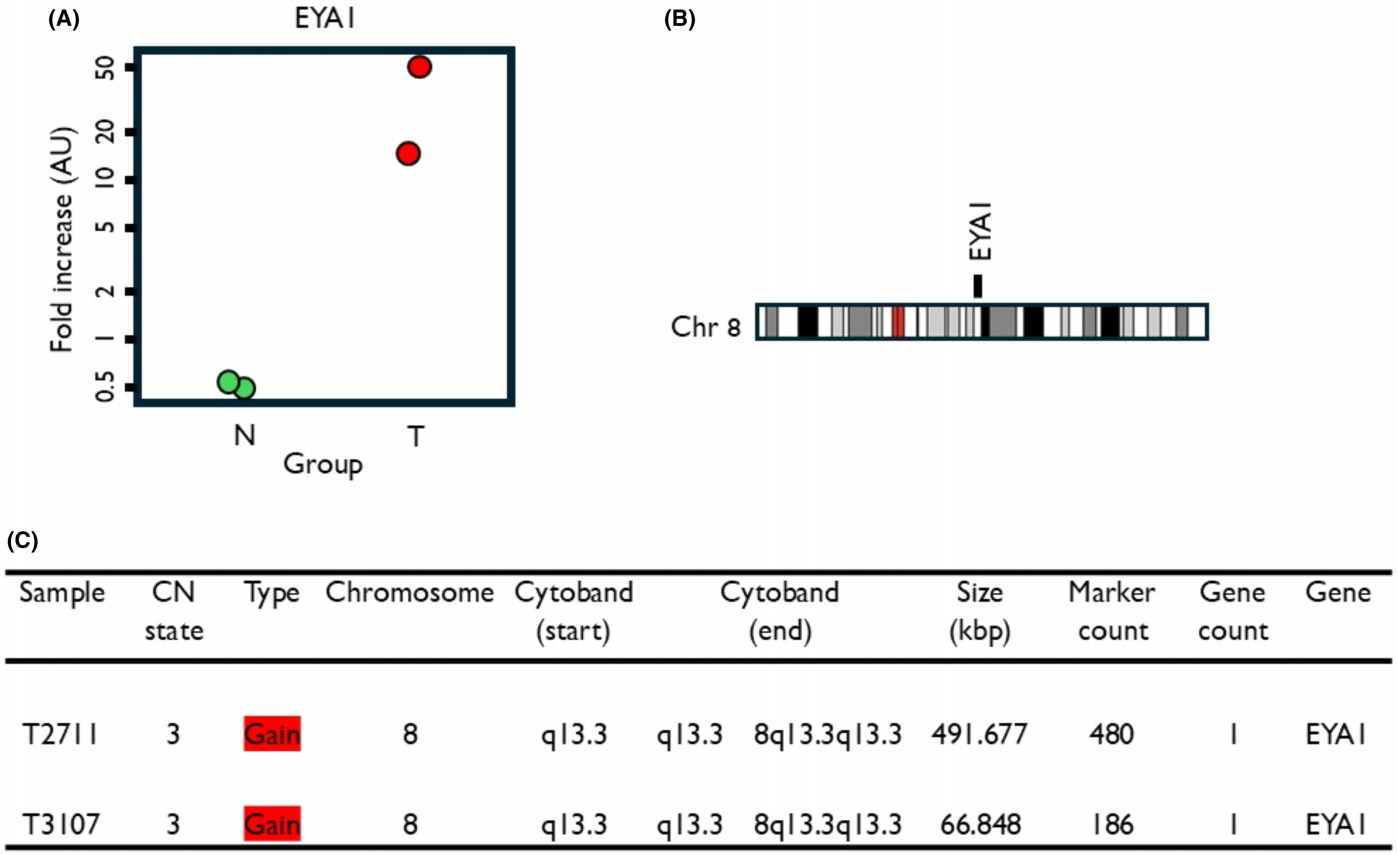
(A) EYA Transcriptional Coactivator And Phosphatase 1 (EYA1) gene expression between the healthy and cancer cell lines and its (B) chromosomal location to the q13.3 band, which showed gains in both cancer samples; (C) detailed characteristics of EYA1.

The 8q13.3 gain region hosts one gene EYA1, which showed significant upregulation in both tumour cell lines. This indicates its role as chromosomal gain and increased expression and its positive correlation with the DEGs in chromosome 8 (Fig. 8). This gene encodes a member of the eyes absent (EYA, Fig. 8A–C) family of proteins that play a role in the developing kidney. Its putative orthlogue in mice can act as a transcriptional activator. In Homo sapiens, mutations in EYAs or disruption of the SIX/EYA complex can cause branchio‐oto‐renal (BOR) syndrome, an autosomal dominant genetic disorder marked by undeveloped or absent kidneys [39]. The sine oculis homeobox homologue 1‐eyes absent homologue 1 (SIX1‐EYA1) transcriptional complex significantly contributes to the pathogenesis of multiple cancers by mediating the expression of genes involved in different biological processes, such as cell cycle progression and metastasis [40].

Overall, this analysis provides mechanistic insight into how genomic structural changes translate into transcriptional outcomes. It bridges cytogenetic aberrations with functional gene expression consequences, helping to distinguish driver alterations from likely passenger events and guiding downstream functional validation or therapeutic target discovery.

### 3.9 Normal and tumour‐derived cells show transcriptionally active HPV

**Fig. 9:**
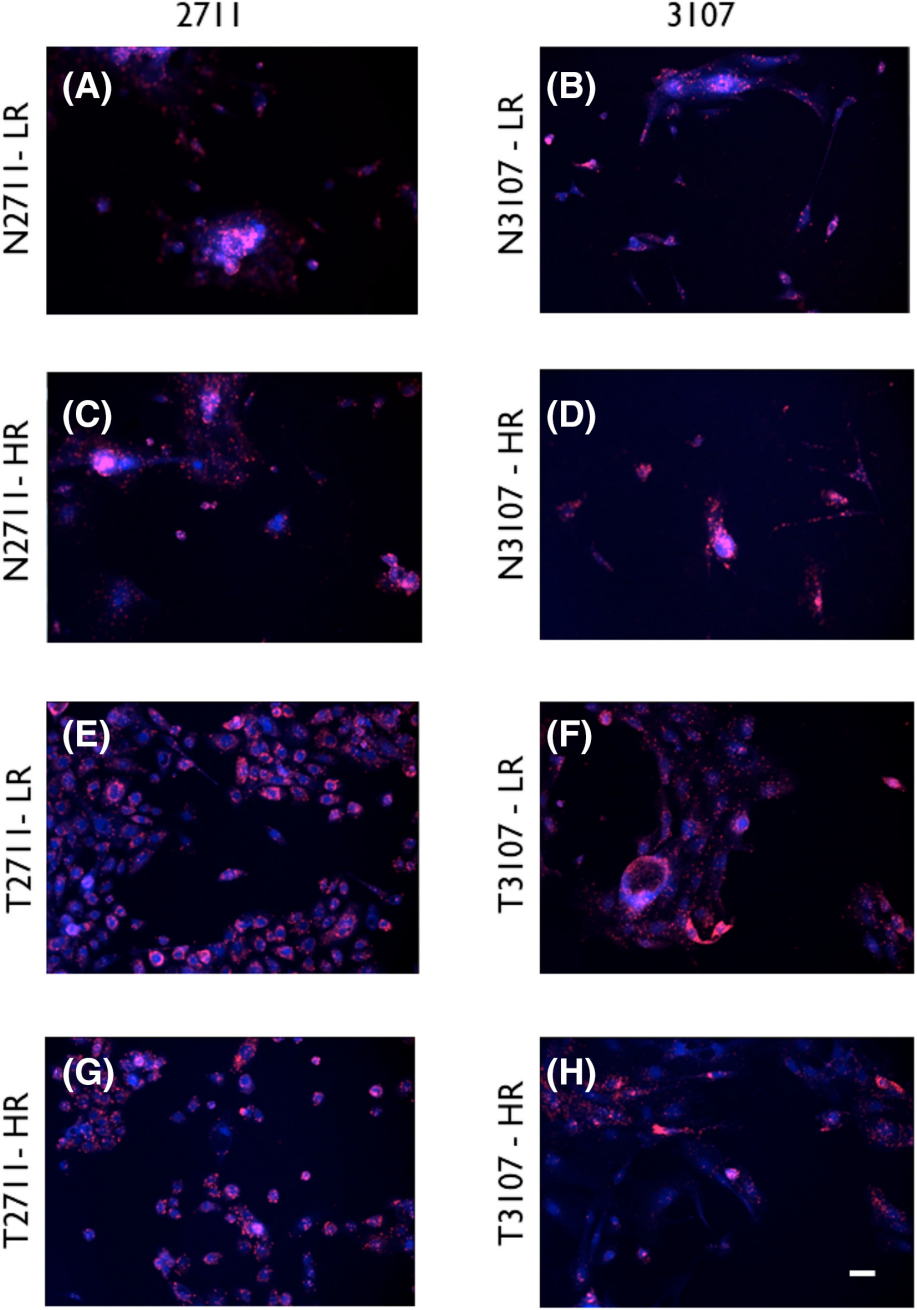
RNAscope analysis of transcriptionally active HPV in normal and tumour cells. Human papillomavirus low‐risk (HPV LR10) and high‐risk (HPV HR18) probes were used that detect 28 different HPV types. Both normal (N2711 and N3107, A–D) and tumour (T2711 and T3107, E–H) were tested for HPV‐LR and HPV‐HR and all appear to express HPV RNA (fuschia); DAPI (4′‐6‐diamidino‐2‐phenylindole, blue) was used as a nuclear stain; scale bar = 10 μm.

We analysed the status of HPV in the cell lines using the immunocytochemical technique of single‐molecule RNA detection (Fig. 9). Both normal (Fig. 9A–D) and tumor cells (Fig. 9E–H) showed distinct, spotted staining associated with RNAscope, indicating the presence of transcriptionally active HPV in the cells.

## 4 Discussion

Penile cancer has remained relatively neglected in terms of scientific investigations partly because of its rarity, particularly in developed countries. As a result, few laboratory tools exist to investigate carcinogenesis mechanisms or test novel therapies. A limited number of penile SCC lines have been established so far [8, 9, 10, 11, 12, 13], and even fewer have undergone full molecular characterisation [10, 13]. New reliable tools need to be established to develop further research regarding this malignancy. Here we established and characterised paired cell lines from normal and tumour tissue from the penis of two penile cancer patients for the first time. This paired samples approach suppresses a level of bias, given that the analysed material comes from the same individual.

We further characterised these cells by using immunochemistry for known squamous cell markers and cytological techniques. Comprehensive molecular analysis for chromosomal aberrations and transcriptomic profiling were performed to identify shared pathways [41] between the cancer lines.

We conducted detailed protein expression analysis of the normal and tumour tissue‐derived lines using markers of squamous epithelium. Our results showed higher expression of basal markers. Similar findings have been described in SCC of skin, head and neck, and cervix. In normal squamous epithelium, proliferative cells attach to basement membrane by integrin class adhesion molecules and express specific keratins like K5 and K14 [42]. Integrins directly regulate proliferation by activating the MAP‐kinase signalling pathway [43], which is in line with our data showing the upregulation of ITGB1 and proliferation‐promoting pathways, like Wnt‐signalling, in cancer cells.

Differentiation markers were also significantly altered in our paired cell lines. However, we did not test how experimental induction of cell differentiation would have affected the expression of these markers. During the normal terminal differentiation process of squamous epithelium, keratinocytes withdraw from the cell cycle and start to express markers of supra‐basal layers. Some of them are keratins, like K10 and K16 [33], while others are structural components of stratified epithelium, like IVL, or other proteins that contribute to the formation of cornified layers, like cross‐link forming enzyme TGM1 [41, 44]. It is well known that the normal differentiation process is disturbed due to the carcinogenic process, which is also supported by our data [44].

Chromosomal rearrangement is a common feature of many cancers and could be important for the aetiology of specific cancers [45]. For example, loss or deletion of chromosome 3p is a common feature in small and nonsmall cell lung carcinoma, an observation associated with loss of tumour suppressor genes [46]. There have been very few systematic investigations on the genomic or proteomic analysis of penile SCC [10, 13]. However, two such studies suggest that Wnt signalling may play a role in penile SCC [1, 47]. It should be noted however that one study reported dysregulation of Wnt targets in the absence of Wnt pathway activation in HPV‐positive penile SCC [9].

In our analysis, gains at 8q13.3 included EYA1 which is a transcriptional coactivator implicated in DNA damage response. Gains at 10q23.2–10q26.13 encompassed multiple genes (GLUD1, SHLD2, WAPL, DMBT1, TACC2, PLEKHA1) associated with genomic stability, DNA‐strand break repair and cell cycle checkpoint control. Similarly, 12p13.33 contained TEAD4 and TULP3, transcriptional regulators linked to oncogenic signalling. Alterations at 20q13.33, a region frequently amplified in other epithelial cancers [48], included PSMA7, SS18L1 and NTSR1, genes known to regulate proteasome activity, chromatin dynamics and neurotrophic signalling [49]. The shared gain at Xq21.1 was notable for encompassing ATRX, ATP7A, PGK1 and LPAR4, all of which have roles in chromatin regulation, metabolism and signal transduction. Finally, both tumour lines exhibited a consistent loss at Yq11.23, affecting multiple testis‐specific genes (DAZ family, BPY2, CDY1), which may reflect structural instability of the Y chromosome in male cancers.

Compared to previous reports of penile cancer cytogenetics, which described recurrent gains on 3p, 8q and 9p and losses on 13q and 18q [50], the current findings show distinct and recurrent copy number alterations. Gains in chromosome 10 and 20 in our analysis highlight novel loci not previously emphasised in PSCC, suggesting potential population‐specific patterns and unique tumour‐intrinsic drivers. These cytoband alterations may provide new insights into the structural genomic instability of penile carcinoma and highlight candidate genes for future mechanistic and therapeutic investigation.

Interestingly, the KEGG pathway analysis identified the disease‐related proteoglycans as a key dysregulated element (Fig. 4). Proteoglycans modulate the expression and activity of cytokines, chemokines, growth factors and adhesion molecules at cell surfaces and function as signalling coreceptors [51]. Furthermore, proteoglycans have a distinct spatial localisation to correct structural development, organisation, hydration and functional properties in normal skin [52]. In addition, proteoglycans connect the cell membrane and the surrounding ECM to regulate and control cancer cell adhesion and migration [53].

On the other hand, aberrant functions of proteoglycans can contribute substantially to the cancer stem cells (CSC) phenotype and therapeutic resistance [54]. One of these proteoglycans that showed alteration in our samples is HSPGs which can function as receptors of cancer cell‐derived exosomes and several viruses [55]. This may be explained by the enrichment of immune‐related pathways and those related to viral infections. It is worth highlighting a few targets identified in our experiments in penile cancer as these are either commonly dysregulated genes in many other cancers or are biomarkers for survival or cancer therapy effectiveness.

A comparative analysis of our data with publicly available GO resource shows the different functions, processes and components that are enriched in our dataset (Fig. 5). The results of this analysis show putative activation of, for example, interferon signalling and lymphocytes and also identified a predicted proportion of immune cells in each sample (Fig. 5). Incidentally, this analysis also shows the inherent differences between the two samples used in this study. This is expected and also cautions against overinterpreting data based on a limited number of samples. Although this is a limitation of our study, the procedures used to develop human models of rare diseases are dogged with such limitations and do not negatively reflect upon the material and knowledge produced here, for the first time for penile cancer.

The top 20 differentially expressed genes identified in tumour samples compared to adjacent healthy penile tissue in our study were enriched in immune and epithelial‐related biological processes. GO analysis highlighted strong activation of interferon alpha/beta signalling, lymphocyte activation and cornified envelope formation. For example, genes such as IFI6, IFIT1, MX1, XAF1, IFNK and LCP1 were associated with interferon‐driven immune signalling, while DSG1, KLK5, KLK8 and KRTDAP were linked to epithelial differentiation and barrier integrity (Fig. 5A,B). Tumour samples also exhibited significantly higher CD4+ T‐cell infiltration (Fig. 5C), consistent with an inflamed tumour microenvironment.

We also compared our DEGs with four published transcriptomic studies in penile cancer [56, 57, 58, 59]. While none of these studies reported overlapping individual genes from our list of top 20 DEGs, they all demonstrated consistent enrichment of the same key pathways: interferon response, immune activation and epithelial tissue remodelling. Our DEGs, while novel, participate in these shared processes, reinforcing their biological relevance. Several of these genes also have known roles in cancer biology. MX1, IFI6, IFIT1 and XAF1 are interferon‐stimulated genes involved in antiviral immunity and apoptosis. KLK5 and KLK8 are serine proteases that promote extracellular matrix remodelling and tumour invasion. ZIC2 is a transcription factor linked to tumour stemness and metastasis, and GRIN2D was previously reported as mutated and prognostically significant in penile cancer [57]. Taken together, this comparison confirms that our study uncovers a novel set of DEGs with high biological relevance, enabled by the unique inclusion of paired tumour and adjacent normal tissue, an experimental design not fully adopted in prior work. It should be noted that these kinds of comparisons have limitations as these are retrospective, performed on different platforms and may have a limited number or incomplete samples (e.g. no normal samples [57]). This supports the idea that, although individual gene‐level changes may vary across cohorts and platforms, the core biological programmes dysregulated in penile carcinogenesis are similar.

Although our DEG set did not overlap directly with earlier reports, both the expression‐ and CNV‐level analyses converge on immune modulation, DNA repair and epithelial remodelling as recurring hallmarks of penile squamous cell carcinoma. The novelty of our dataset lies in identifying previously unreported cytoband‐level alterations (notably at 10q and 20q) that complement and extend the transcriptomic evidence from earlier studies, highlighting population‐specific and tumour‐intrinsic mechanisms.

There are many difficulties in making cell lines for rare diseases and more so for cell lines that are paired representatives of normal and cancer from the same patient. It is known that there are changes to the genetic landscape when cells are propagated from tissue [60]. It ought to be noted that a large number of cell lines do reflect the genetics of cancer they are derived from [60]. Another limitation in our study is the lack of genetic profiling of the original tissue. However, this is a general gap in our knowledge in this field. For example, COSMIC (https://cancer.sanger.ac.uk/cosmic) is one of the largest database for mutations in hundreds of thousands of cancer samples from all types of tissue. Unfortunately, there are only around 650 tissue samples for penile carcinoma, this is in contrast to breast tissue for which there are over 57 000 samples.

## 5 Conclusions

Our results constitute an important step forward in the development of tools to discover putative cellular mechanisms involved in penile carcinogenesis and disease progression. Penile‐preserving procedures are now used more commonly than penile amputation for the primary tumour, reducing physical and psychological consequences. However, the outcomes for patients with metastatic disease remain poor due to ineffective chemotherapy regimens. To develop novel therapies, this *in vitro* model may now offer a means to investigate targeted therapies.

## Conflict of interest

The authors declare no conflict of interest.

## Author contributions

AA and AM conceived and designed the study and secured funding; SB, MH, TB, KP, SK, NB, KS and MR performed the experiments; AM, MH, SB, TB, MA and R Hamoudi and R Henrique conducted the analysis; SB, MH, TB, RH, and AA interpreted the data; AA and MH wrote the manuscript; AA, AM, SB, MH and KS edited the manuscript; all authors reviewed the manuscript.

## Supporting information


**Data S1.** A Python Script.
**Data S2**. A table of PCR primers.
**Data S3**. A table of STR profiles.

## Data Availability

Gene expression data presented in this manuscript is available upon request from the authors.
