# Supplementary material for: Molecular characterisation of human penile carcinoma and generation of paired epithelial primary cell lines
Source: Mol Oncol. 2025 Nov 25;20(2):260–81. doi: 10.1002/1878-0261.70156 (PMC12936413; doi:10.1002/1878-0261.70156)
Supplement: Supplementary file 1 — Data S1. A Python Script. Data S2. A table of PCR primers. Data S3. A table of STR profiles. [file MOL2-20-260-s001.zip › Data S1-S3.docx]

**Supporting Information**

***Supplementary Data S1: Python Script***

*Python code used for fluorescence quantitation in this study*

**from** PIL **import** Image

**import** glob

**import** operator

*# load images and turn the blue channel off*

path = 'C:/Users/AAGroup/Desktop/Aamir'

filenames = glob.glob(path + '/*.jpg')

**for** filename **in** filenames:

n = Image.open(filename)

m = n.load()

s = n.size

**for** x **in** range(s[0]):

**for** y **in** range(s[1]):

r,g,b = m[x,y]

m[x,y] = r,g,0

n.save(filename+'blue_off.jpg', "JPEG")

*# load images with blue channel off and count green pixels*

filenames2 = glob.glob(path + '/*blue_off.jpg')

**for** filename **in** filenames2:

x = Image.open(filename).getdata()

green_I = 0

**for** pixel **in** list(x):

red, green, blue = pixel

**if** green > 15: *#threshold applied to remove the noise*

green_I += 1

print(filename)

print(green_I)

***Supplementary Data S2: Table of Polymerase chain reaction (PCR) primers***

*PCR primers used in this study*

| Gene | Forward |  |  | Reverse |  |  | Product (bp) |
| --- | --- | --- | --- | --- | --- | --- | --- |
| Ecad | TGAGCTCCCTGACAAAAATA | | | AGGTACCACATTCGTCACTG | | | 457 |
| K14 | TACCTGAAGAAGAACCACGA | | | AGGAGGTCACATCTCTGGAT | | | 583 |
| ITGB1 | TGTAACCAACCGTAGCAAAG | | | ATCAGTGATCCACAAACTGC | | | 513 |
| K5 | GGAGGTATCCAAGAGGTCAC | | | GACCACTGAGGTGTCAGAGA | | | 561 |
| K16 | GGACCAAGTATGAGCATGAA | | | GATCTGGTACTCCTGGCTCT | | | 599 |
| K10 | TCGTGGAAGCTATGGAAGTA | | | CTCTCAATTTGCATCTCCAG | | | 570 |
| IVL | CAAGAGAGATGAGCAACTGG | | | GAGCTCTGGCTGCTTCTC | | | 499 |
| TGM1 | CTTTCATTTTTGCTGAGGTG | | | CTCCACTTCCTTCTTGGTCT | | | 437 |

***Supplementary Data S3: Table of Short tandem repeat (STR) profiles***

*STR analysis of two paired penile cancer cell lines*

**N2711**

| *Marker* | *Allele 1* | *Allele 2* |
| --- | --- | --- |
| AMEL | X | Y |
| CSF1PO | 11 | 11 |
| D13S317 | 11 | 12 |
| D16S539 | 12 | 12 |
| D18S51 | 12 | 12 |
| D21S11 | 31.2 | 32.2 |
| D3S1358 | 14 | 17 |
| D5S818 | 10 | 11 |
| D7S820 | 12 | 14 |
| D8S1179 | 12 | 13 |
| FGA | 23 | 24 |
| Penta D | 10 | 12 |
| Penta E | 7 | 13 |
| TH01 | 6 | 9 |
| TPOX | 11 | 11 |
| vWA | 15 | 20 |

**N3107**

| *Marker* | *Allele 1* | *Allele 2* |
| --- | --- | --- |
| AMEL | X | Y |
| CSF1PO | 10 | 11 |
| D13S317 | 9 | 12 |
| D16S539 | 9 | 13 |
| D18S51 | 16 | 17 |
| D21S11 | 30 | 31 |
| D3S1358 | 15 | 18 |
| D5S818 | 11 | 11 |
| D7S820 | 8 | 12 |
| D8S1179 | 13 | 14 |
| FGA | 18 | 24 |
| Penta D | 12 | 13 |
| Penta E | 7 | 18 |
| TH01 | 7 | 8 |
| TPOX | 8 | 9 |
| vWA | 15 | 17 |
|  |  |  |

**T2711**

| *Marker* | *Allele 1* | *Allele 2* |
| --- | --- | --- |
| AMEL | X | Y |
| CSF1PO | 11 | 11 |
| D13S317 | 11 | 12 |
| D16S539 | 12 | 12 |
| D18S51 | 12 | 12 |
| D21S11 | 31.2 | 32.2 |
| D3S1358 | 14 | 14 |
| D5S818 | 10 | 11 |
| D7S820 | 12 | 14 |
| D8S1179 | 12 | 13 |
| FGA | 23 | 24 |
| Penta D | 10 | 12 |
| Penta E | 7 | 13 |
| TH01 | 6 | 9 |
| TPOX | 11 | 11 |
| vWA | 15 | 20 |

**T3107**

| *Marker* | *Allele 1* | *Allele 2* |
| --- | --- | --- |
| AMEL | X | Y |
| CSF1PO | 10 | 11 |
| D13S317 | 9 | 12 |
| D16S539 | 9 | 13 |
| D18S51 | 16 | 17 |
| D21S11 | 30 | 31 |
| D3S1358 | 15 | 18 |
| D5S818 | 11 | 11 |
| D7S820 | 8 | 12 |
| D8S1179 | 13 | 14 |
| FGA | 18 | 24 |
| Penta D | 12 | 13 |
| Penta E | 7 | 18 |
| TH01 | 7 | 8 |
| TPOX | 8 | 9 |
| vWA | 15 | 17 |
