# Supplementary material for: Molecular characterisation of human penile carcinoma and generation of paired epithelial primary cell lines
Source: Mol Oncol. 2025 Nov 25;20(2):260–81. doi: 10.1002/1878-0261.70156 (PMC12936413; doi:10.1002/1878-0261.70156)
Supplement: Supplementary file 1 — Data S1. A Python Script. Data S2. A table of PCR primers. Data S3. A table of STR profiles. [file MOL2-20-260-s001.zip › Broad_et_al_Supplementary.pdf]

Supplementary Data – S1

Python code for fluorescence quantitation

```
from PIL import Image
import glob
import operator
# load images and turn the blue channel off
path = 'C:/Users/AAGroup/Desktop/Aamir'
filenames = glob.glob(path + '/*.jpg')
for filename in filenames:
n = Image.open(filename)
m = n.load()
s = n.size
for x in range(s[0]):
for y in range(s[1]):
r,g,b = m[x,y]
m[x,y] = r,g,0
n.save(filename+'blue_off.jpg', "JPEG")
# load images with blue channel off and count green pixels
filenames2 = glob.glob(path + '/*blue_off.jpg')
for filename in filenames2:
x = Image.open(filename).getdata()
green_I = 0
for pixel in list(x):
red, green, blue = pixel
if green > 15: #threshold applied to remove the noise
green_I += 1
print(filename)
print(green_I)
```

Supplementary Table S2 : PCR primers

Gene	Forward	Reverse	Product (bp)
Ecad	TGAGCTCCCTGACAAAAATA	AGGTACCACATTCGTCACTG	457
K14	TACCTGAAGAAGAACCACGA	AGGAGGTCACATCTCTGGAT	583
ITGB1	TGTAACCAACCGTAGCAAAG	ATCAGTGATCCACAAACTGC	513
K5	GGAGGTATCCAAGAGGTCAC	GACCACTGAGGTGTCAGAGA	561
K16	GGACCAAGTATGAGCATGAA	GATCTGGTACTCCTGGCTCT	599
K10	TCGTGGAAGCTATGGAAGTA	CTCTCAATTTGCATCTCCAG	570
IVL	CAAGAGAGATGAGCAACTG<br>G	GAGCTCTGGCTGCTTCTC	499
TGM1	CTTTCATTTTTGCTGAGGTG	CTCCACTTCCTTCTTGGTCT	437

Supplementary Table S3

N2711

Marker	*Allele 1*	*Allele 2*
AMEL	X	Y
CSF1PO	11	11
D13S317	11	12
D16S539	12	12
D18S51	12	12
D21S11	31.2	32.2
D3S1358	14	17
D5S818	10	11
D7S820	12	14
D8S1179	12	13
FGA	23	24
Penta D	10	12
Penta E	7	13
TH01	6	9
TPOX	11	11
vWA	15	20

T2711

Marker	*Allele 1*	*Allele 2*
AMEL	X	Y
CSF1PO	11	11
D13S317	11	12
D16S539	12	12
D18S51	12	12
D21S11	31.2	32.2
D3S1358	14	14
D5S818	10	11
D7S820	12	14
D8S1179	12	13
FGA	23	24
Penta D	10	12
Penta E	7	13
TH01	6	9
TPOX	11	11
vWA	15	20

N3107

Marker	*Allele 1*	*Allele 2*
AMEL	X	Y
CSF1PO	10	11
D13S317	9	12
D16S539	9	13
D18S51	16	17
D21S11	30	31
D3S1358	15	18
D5S818	11	11
D7S820	8	12
D8S1179	13	14
FGA	18	24
Penta D	12	13
Penta E	7	18
TH01	7	8
TPOX	8	9
vWA	15	17

T3107

Marker	*Allele 1*	*Allele 2*
AMEL	X	Y
CSF1PO	10	11
D13S317	9	12
D16S539	9	13
D18S51	16	17
D21S11	30	31
D3S1358	15	18
D5S818	11	11
D7S820	8	12
D8S1179	13	14
FGA	18	24
Penta D	12	13
Penta E	7	18
TH01	7	8
TPOX	8	9
vWA	15	17

Short tandem repeat (STR) analysis of two paired penile cancer cell lines.
